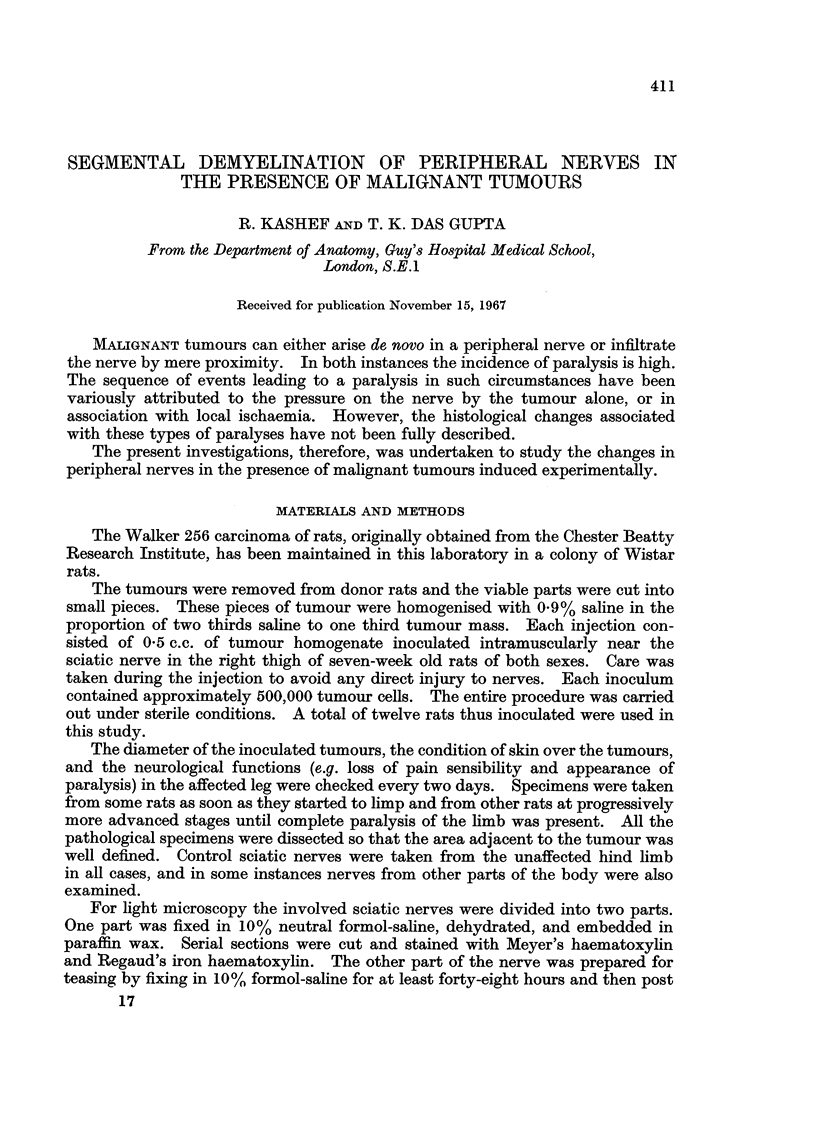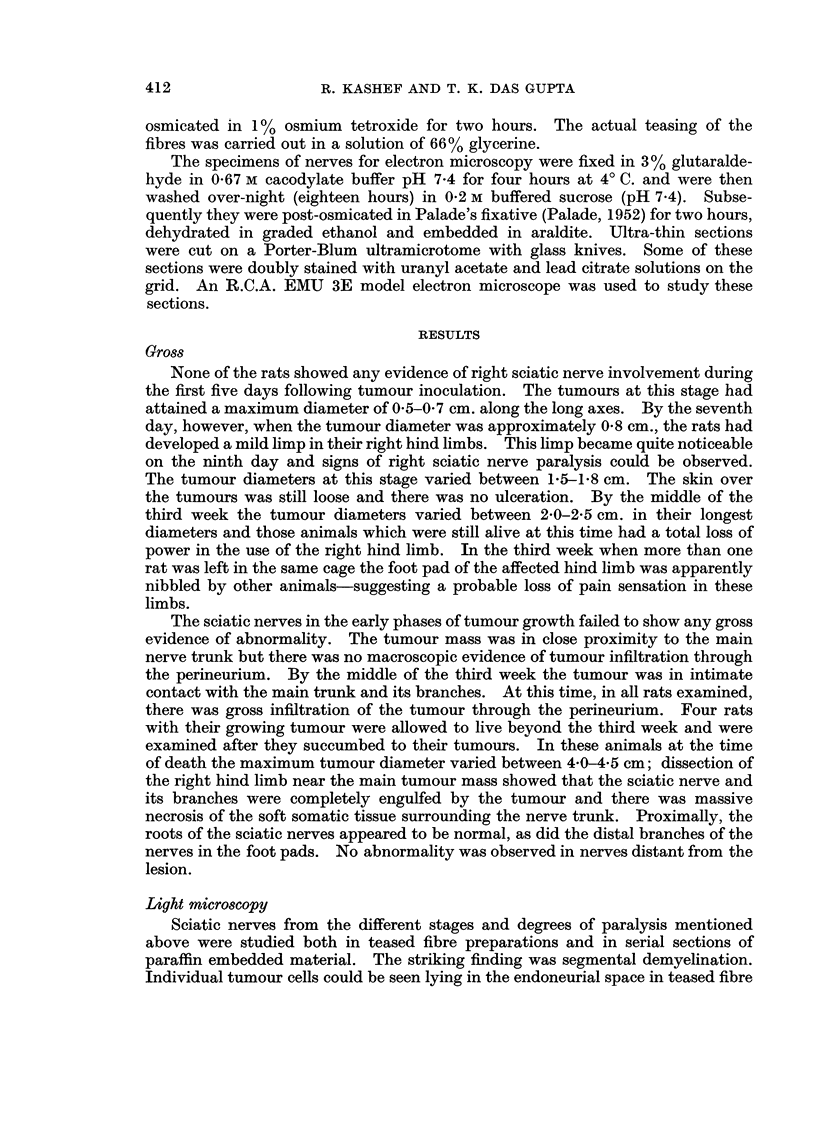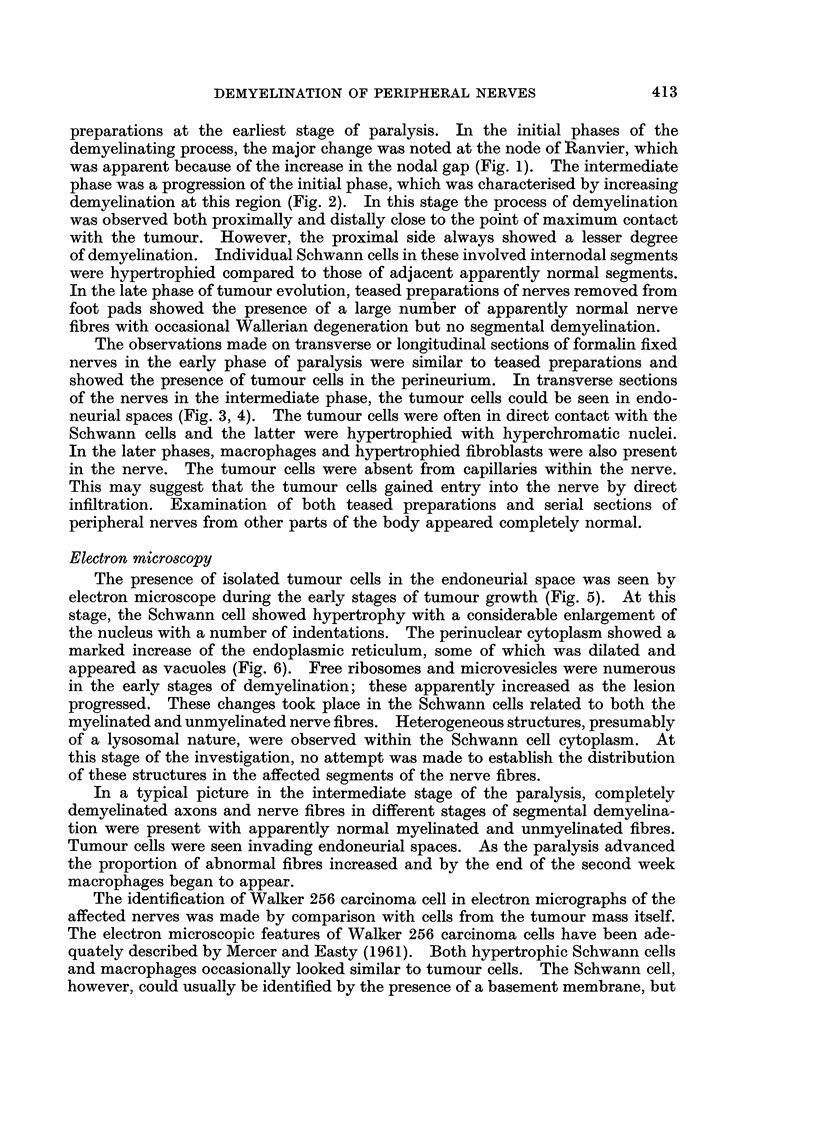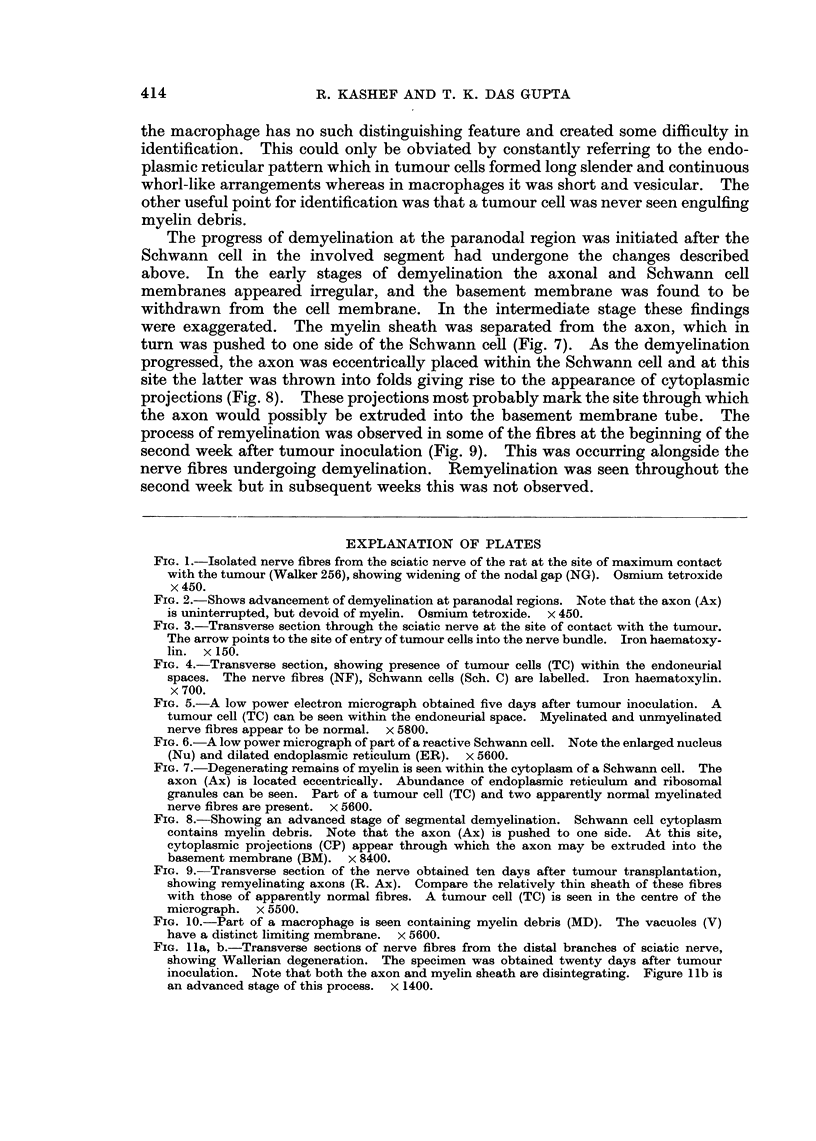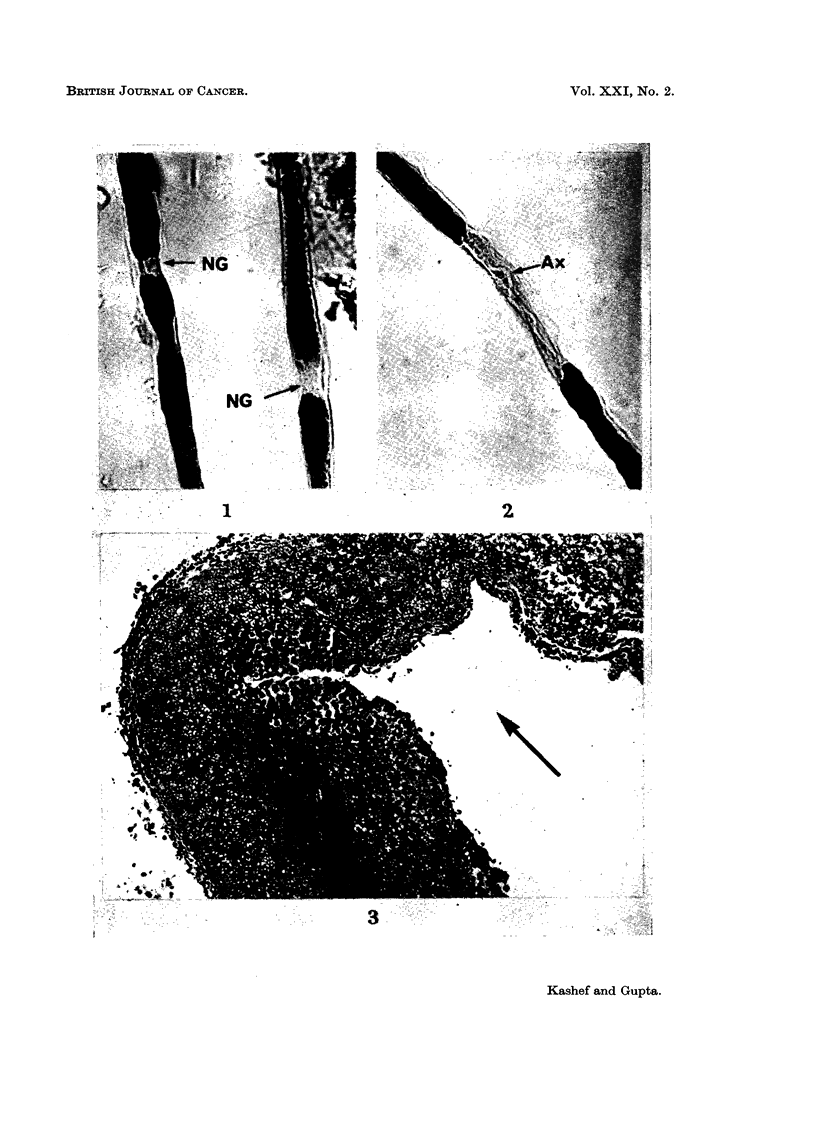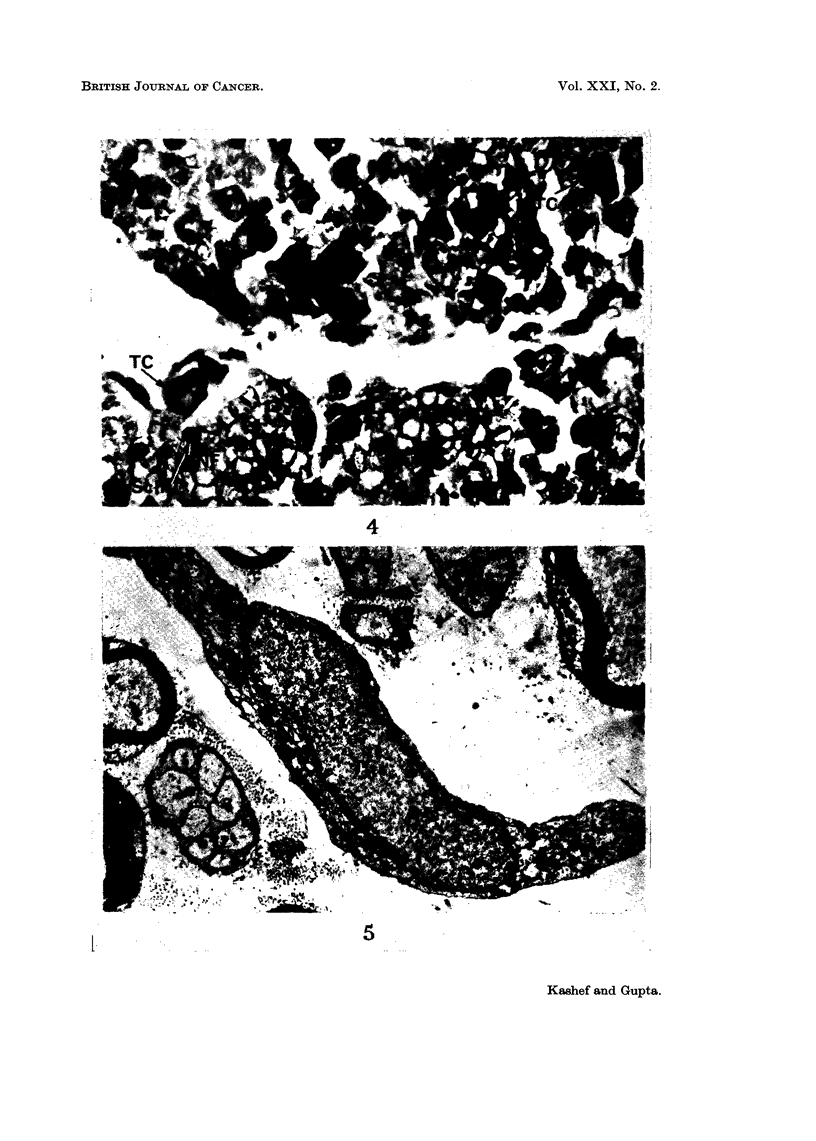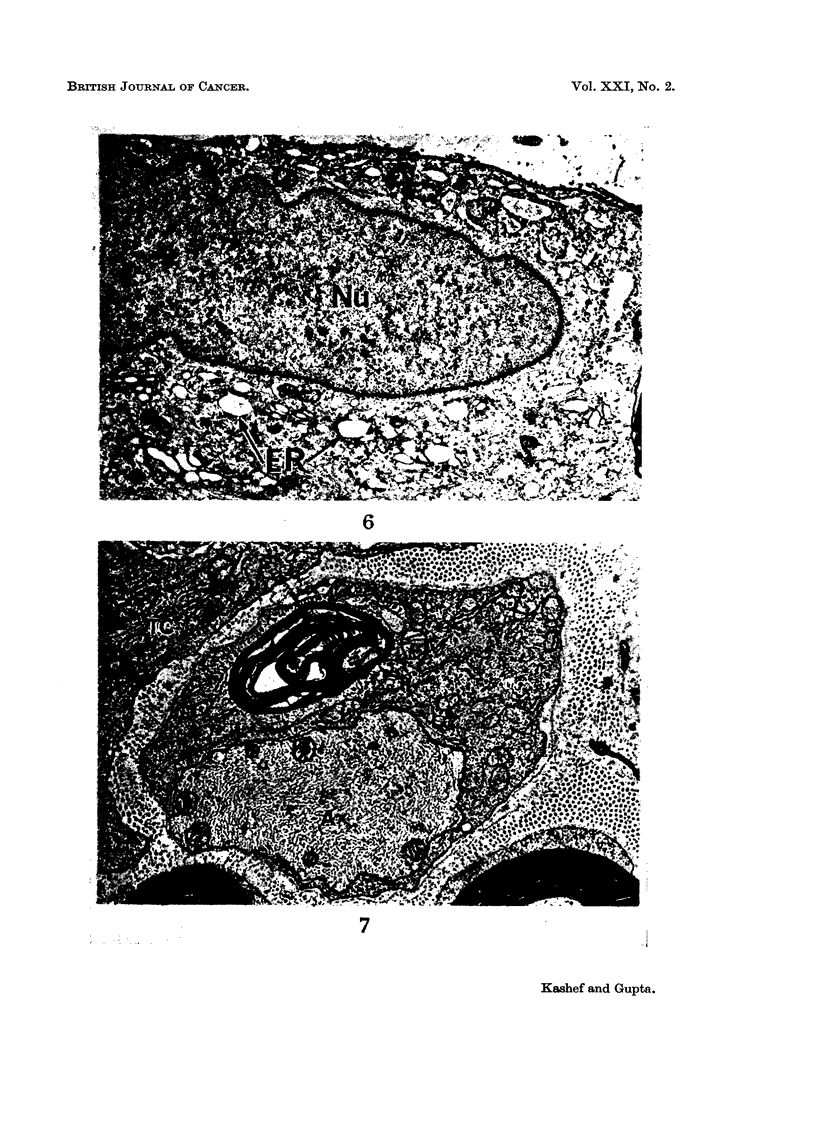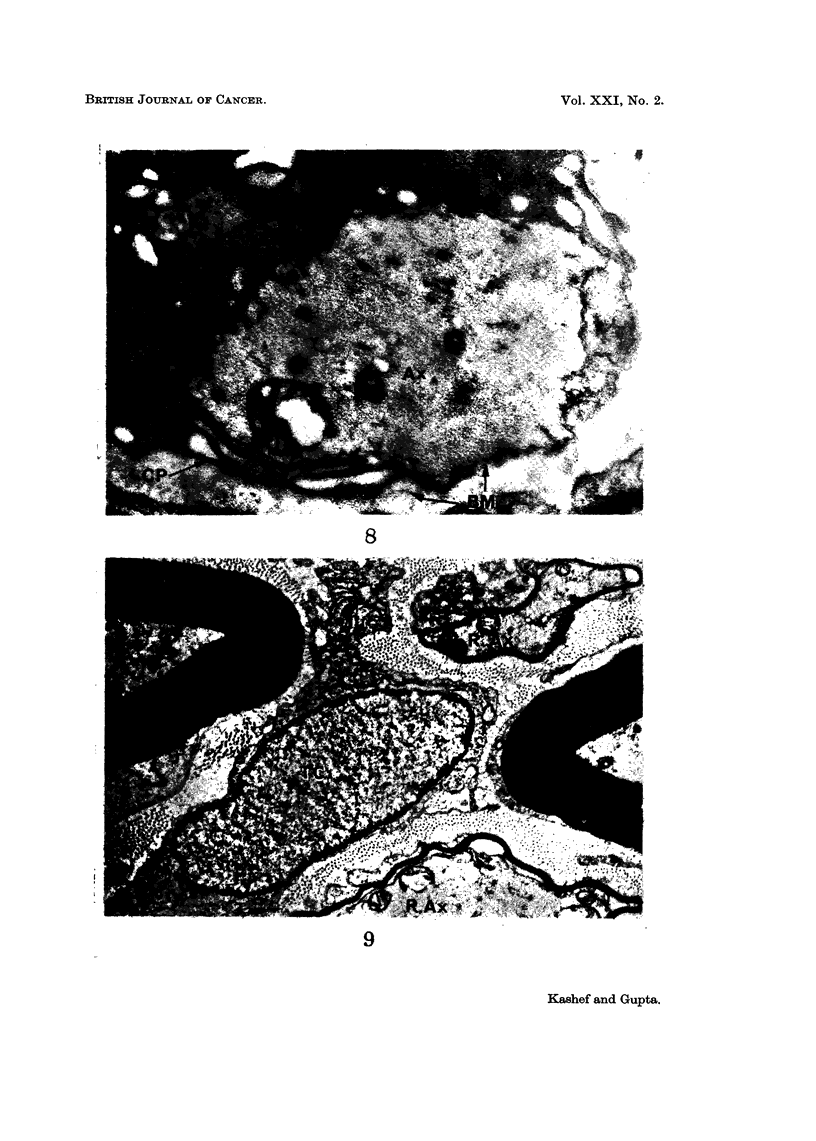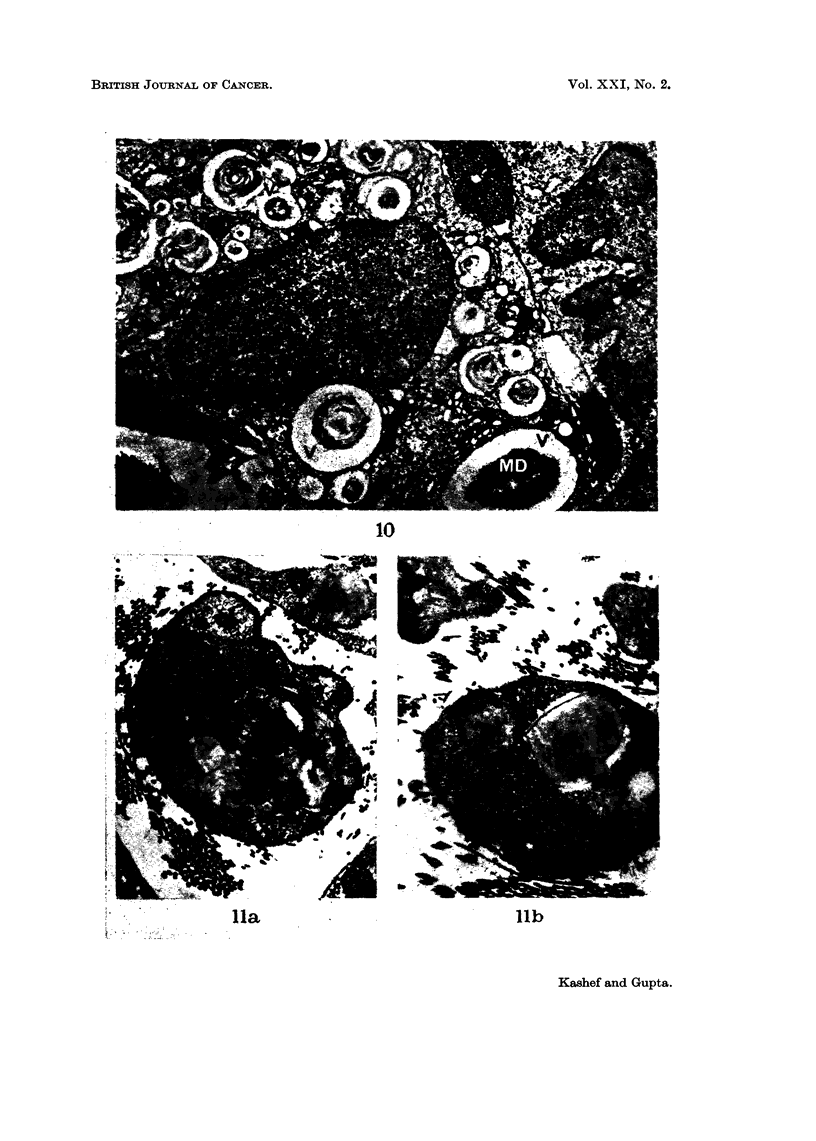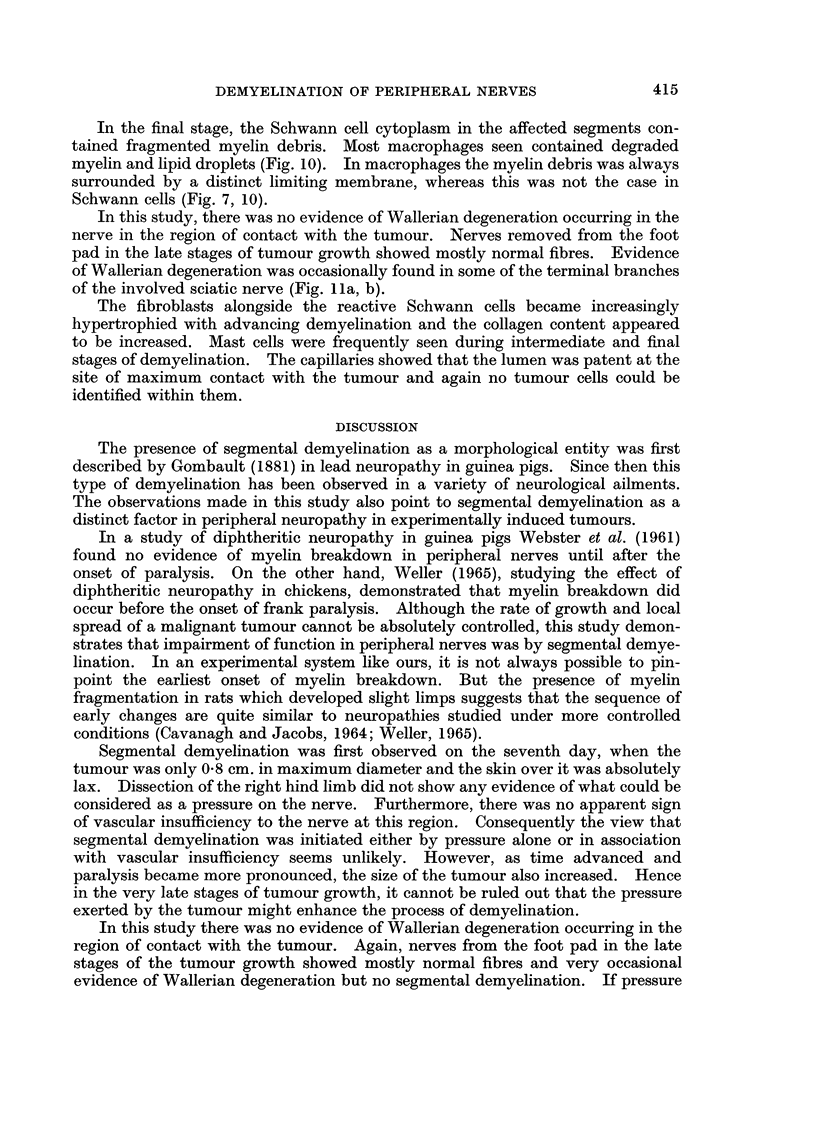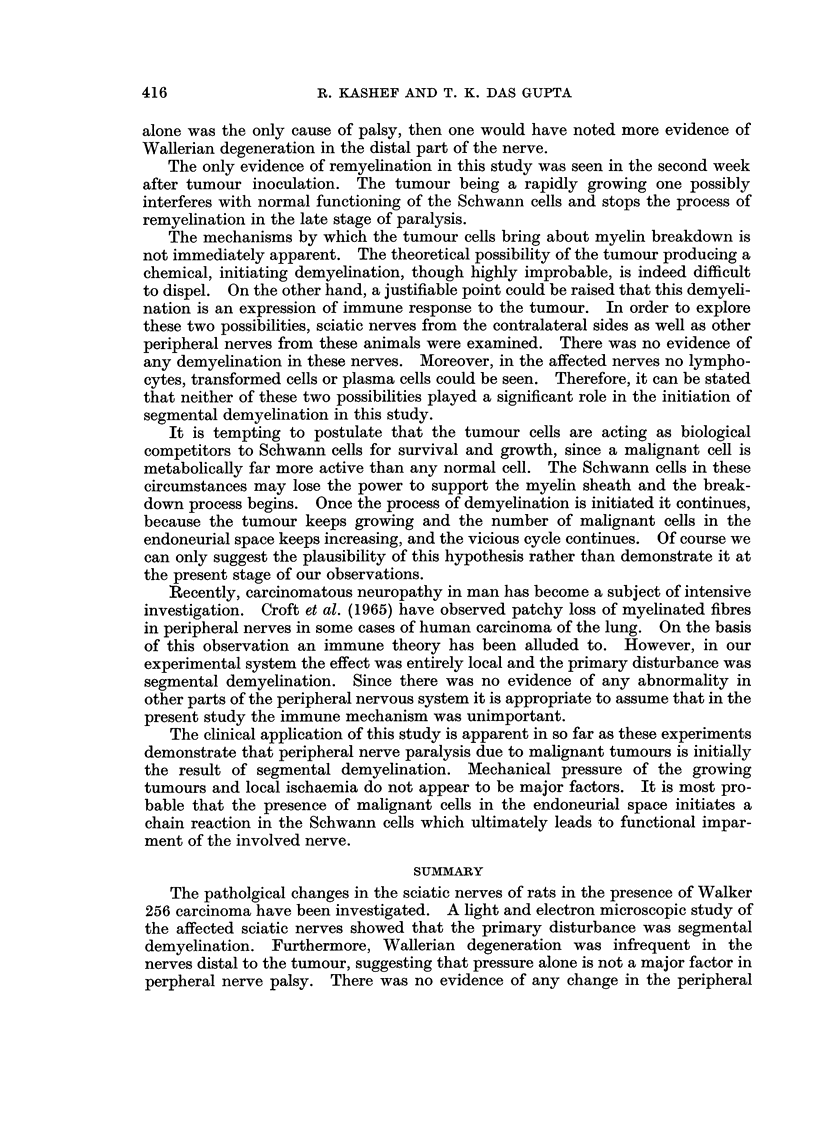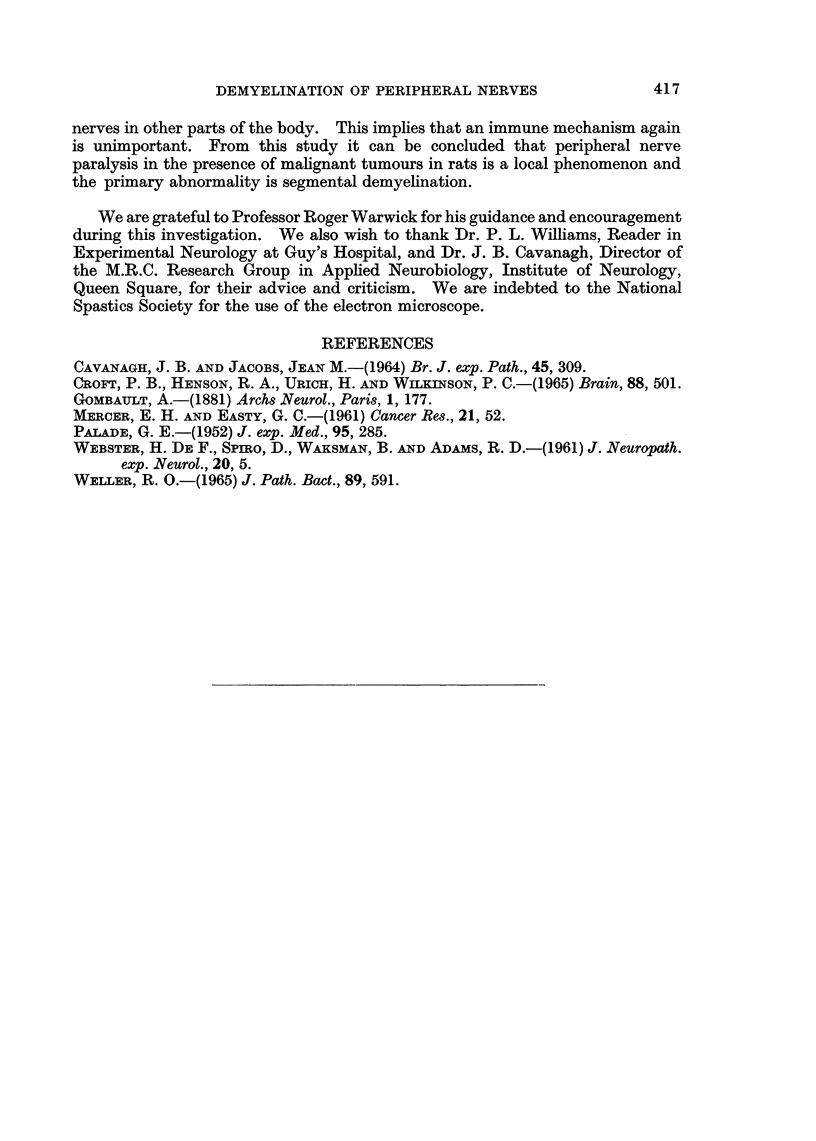# Segmental demyelination of peripheral nerves in the presence of malignant tumours.

**DOI:** 10.1038/bjc.1967.47

**Published:** 1967-06

**Authors:** R. Kashef, T. K. Das Gupta

## Abstract

**Images:**


### SEGMENTAL DEMYELINATION OF PERIPHERAL NERVES IN THE PRESENCE OF MALIGNANT TUMOURS

R. KASHEF AND T. K. DAS GUPTA

*From the Department of Anatomy, Guy's Hospital Medical School, London, S.E.1*

Received for publication November 15, 1967

MALIGNANT tumours can either arise *de novo* in a peripheral nerve or infiltrate the nerve by mere proximity. In both instances the incidence of paralysis is high. The sequence of events leading to a paralysis in such circumstances have been variously attributed to the pressure on the nerve by the tumour alone, or in association with local ischaemia. However, the histological changes associated with these types of paralyses have not been fully described.

The present investigations, therefore, was undertaken to study the changes in peripheral nerves in the presence of malignant tumours induced experimentally.

### MATERIALS AND METHODS

The Walker 256 carcinoma of rats, originally obtained from the Chester Beatty Research Institute, has been maintained in this laboratory in a colony of Wistar rats.

The tumours were removed from donor rats and the viable parts were cut into small pieces. These pieces of tumour were homogenised with 0·9% saline in the proportion of two thirds saline to one third tumour mass. Each injection consisted of 0·5 c.c. of tumour homogenate inoculated intramuscularly near the sciatic nerve in the right thigh of seven-week old rats of both sexes. Care was taken during the injection to avoid any direct injury to nerves. Each inoculum contained approximately 500,000 tumour cells. The entire procedure was carried out under sterile conditions. A total of twelve rats thus inoculated were used in this study.

The diameter of the inoculated tumours, the condition of skin over the tumours, and the neurological functions (*e.g.* loss of pain sensibility and appearance of paralysis) in the affected leg were checked every two days. Specimens were taken from some rats as soon as they started to limp and from other rats at progressively more advanced stages until complete paralysis of the limb was present. All the pathological specimens were dissected so that the area adjacent to the tumour was well defined. Control sciatic nerves were taken from the unaffected hind limb in all cases, and in some instances nerves from other parts of the body were also examined.

For light microscopy the involved sciatic nerves were divided into two parts. One part was fixed in 10% neutral formol-saline, dehydrated, and embedded in paraffin wax. Serial sections were cut and stained with Meyer's haematoxylin and Regaud's iron haematoxylin. The other part of the nerve was prepared for teasing by fixing in 10% formol-saline for at least forty-eight hours and then post osmicated in 1% osmium tetroxide for two hours. The actual teasing of the fibres was carried out in a solution of 66% glycerine.

The specimens of nerves for electron microscopy were fixed in 3% glutaraldehyde in 0·67 M cacodylate buffer pH 7·4 for four hours at 4° C. and were then washed over-night (eighteen hours) in 0·2 M buffered sucrose (pH 7·4). Subsequently they were post-osmicated in Palade's fixative (Palade, 1952) for two hours, dehydrated in graded ethanol and embedded in araldite. Ultra-thin sections were cut on a Porter-Blum ultramicrotome with glass knives. Some of these sections were doubly stained with uranyl acetate and lead citrate solutions on the grid. An R.C.A. EMU 3E model electron microscope was used to study these sections.

### RESULTS

### *Gross*

None of the rats showed any evidence of right sciatic nerve involvement during the first five days following tumour inoculation. The tumours at this stage had attained a maximum diameter of 0·5–0·7 cm. along the long axes. By the seventh day, however, when the tumour diameter was approximately 0·8 cm., the rats had developed a mild limp in their right hind limbs. This limp became quite noticeable on the ninth day and signs of right sciatic nerve paralysis could be observed. The tumour diameters at this stage varied between 1·5–1·8 cm. The skin over the tumours was still loose and there was no ulceration. By the middle of the third week the tumour diameters varied between 2·0–2·5 cm. in their longest diameters and those animals which were still alive at this time had a total loss of power in the use of the right hind limb. In the third week when more than one rat was left in the same cage the foot pad of the affected hind limb was apparently nibbled by other animals—suggesting a probable loss of pain sensation in these limbs.

The sciatic nerves in the early phases of tumour growth failed to show any gross evidence of abnormality. The tumour mass was in close proximity to the main nerve trunk but there was no macroscopic evidence of tumour infiltration through the perineurium. By the middle of the third week the tumour was in intimate contact with the main trunk and its branches. At this time, in all rats examined, there was gross infiltration of the tumour through the perineurium. Four rats with their growing tumour were allowed to live beyond the third week and were examined after they succumbed to their tumours. In these animals at the time of death the maximum tumour diameter varied between 4·0–4·5 cm; dissection of the right hind limb near the main tumour mass showed that the sciatic nerve and its branches were completely engulfed by the tumour and there was massive necrosis of the soft somatic tissue surrounding the nerve trunk. Proximally, the roots of the sciatic nerves appeared to be normal, as did the distal branches of the nerves in the foot pads. No abnormality was observed in nerves distant from the lesion.

### *Light microscopy*

Sciatic nerves from the different stages and degrees of paralysis mentioned above were studied both in teased fibre preparations and in serial sections of paraffin embedded material. The striking finding was segmental demyelination. Individual tumour cells could be seen lying in the endoneurial space in teased fibre preparations at the earliest stage of paralysis. In the initial phases of the demyelinating process, the major change was noted at the node of Ranvier, which was apparent because of the increase in the nodal gap (Fig. 1). The intermediate phase was a progression of the initial phase, which was characterised by increasing demyelination at this region (Fig. 2). In this stage the process of demyelination was observed both proximally and distally close to the point of maximum contact with the tumour. However, the proximal side always showed a lesser degree of demyelination. Individual Schwann cells in these involved internodal segments were hypertrophied compared to those of adjacent apparently normal segments. In the late phase of tumour evolution, teased preparations of nerves removed from foot pads showed the presence of a large number of apparently normal nerve fibres with occasional Wallerian degeneration but no segmental demyelination.

The observations made on transverse or longitudinal sections of formalin fixed nerves in the early phase of paralysis were similar to teased preparations and showed the presence of tumour cells in the perineurium. In transverse sections of the nerves in the intermediate phase, the tumour cells could be seen in endoneurial spaces (Fig. 3, 4). The tumour cells were often in direct contact with the Schwann cells and the latter were hypertrophied with hyperchromatic nuclei. In the later phases, macrophages and hypertrophied fibroblasts were also present in the nerve. The tumour cells were absent from capillaries within the nerve. This may suggest that the tumour cells gained entry into the nerve by direct infiltration. Examination of both teased preparations and serial sections of peripheral nerves from other parts of the body appeared completely normal.

*Electron microscopy*

The presence of isolated tumour cells in the endoneurial space was seen by electron microscope during the early stages of tumour growth (Fig. 5). At this stage, the Schwann cell showed hypertrophy with a considerable enlargement of the nucleus with a number of indentations. The perinuclear cytoplasm showed a marked increase of the endoplasmic reticulum, some of which was dilated and appeared as vacuoles (Fig. 6). Free ribosomes and microvesicles were numerous in the early stages of demyelination; these apparently increased as the lesion progressed. These changes took place in the Schwann cells related to both the myelinated and unmyelinated nerve fibres. Heterogeneous structures, presumably of a lysosomal nature, were observed within the Schwann cell cytoplasm. At this stage of the investigation, no attempt was made to establish the distribution of these structures in the affected segments of the nerve fibres.

In a typical picture in the intermediate stage of the paralysis, completely demyelinated axons and nerve fibres in different stages of segmental demyelination were present with apparently normal myelinated and unmyelinated fibres. Tumour cells were seen invading endoneurial spaces. As the paralysis advanced the proportion of abnormal fibres increased and by the end of the second week macrophages began to appear.

The identification of Walker 256 carcinoma cell in electron micrographs of the affected nerves was made by comparison with cells from the tumour mass itself. The electron microscopic features of Walker 256 carcinoma cells have been adequately described by Mercer and Easty (1961). Both hypertrophic Schwann cells and macrophages occasionally looked similar to tumour cells. The Schwann cell, however, could usually be identified by the presence of a basement membrane, but the macrophage has no such distinguishing feature and created some difficulty in identification. This could only be obviated by constantly referring to the endoplasmic reticular pattern which in tumour cells formed long slender and continuous whorl-like arrangements whereas in macrophages it was short and vesicular. The other useful point for identification was that a tumour cell was never seen engulfing myelin debris.

The progress of demyelination at the paranodal region was initiated after the Schwann cell in the involved segment had undergone the changes described above. In the early stages of demyelination the axonal and Schwann cell membranes appeared irregular, and the basement membrane was found to be withdrawn from the cell membrane. In the intermediate stage these findings were exaggerated. The myelin sheath was separated from the axon, which in turn was pushed to one side of the Schwann cell (Fig. 7). As the demyelination progressed, the axon was eccentrically placed within the Schwann cell and at this site the latter was thrown into folds giving rise to the appearance of cytoplasmic projections (Fig. 8). These projections most probably mark the site through which the axon would possibly be extruded into the basement membrane tube. The process of remyelination was observed in some of the fibres at the beginning of the second week after tumour inoculation (Fig. 9). This was occurring alongside the nerve fibres undergoing demyelination. Remyelination was seen throughout the second week but in subsequent weeks this was not observed.

---

### EXPLANATION OF PLATES

FIG. 1.—Isolated nerve fibres from the sciatic nerve of the rat at the site of maximum contact with the tumour (Walker 256), showing widening of the nodal gap (NG). Osmium tetroxide × 450.

FIG. 2.—Shows advancement of demyelination at paranodal regions. Note that the axon (Ax) is uninterrupted, but devoid of myelin. Osmium tetroxide. × 450.

FIG. 3.—Transverse section through the sciatic nerve at the site of contact with the tumour. The arrow points to the site of entry of tumour cells into the nerve bundle. Iron haematoxylin. × 150.

FIG. 4.—Transverse section, showing presence of tumour cells (TC) within the endoneurial spaces. The nerve fibres (NF), Schwann cells (Sch. C) are labelled. Iron haematoxylin. × 700.

FIG. 5.—A low power electron micrograph obtained five days after tumour inoculation. A tumour cell (TC) can be seen within the endoneurial space. Myelinated and unmyelinated nerve fibres appear to be normal. × 5800.

FIG. 6.—A low power micrograph of part of a reactive Schwann cell. Note the enlarged nucleus (Nu) and dilated endoplasmic reticulum (ER). × 5600.

FIG. 7.—Degenerating remains of myelin is seen within the cytoplasm of a Schwann cell. The axon (Ax) is located eccentrically. Abundance of endoplasmic reticulum and ribosomal granules can be seen. Part of a tumour cell (TC) and two apparently normal myelinated nerve fibres are present. × 5600.

FIG. 8.—Showing an advanced stage of segmental demyelination. Schwann cell cytoplasm contains myelin debris. Note that the axon (Ax) is pushed to one side. At this site, cytoplasmic projections (CP) appear through which the axon may be extruded into the basement membrane (BM). × 8400.

FIG. 9.—Transverse section of the nerve obtained ten days after tumour transplantation, showing remyelinating axons (R. Ax). Compare the relatively thin sheath of these fibres with those of apparently normal fibres. A tumour cell (TC) is seen in the centre of the micrograph. × 5500.

FIG. 10.—Part of a macrophage is seen containing myelin debris (MD). The vacuoles (V) have a distinct limiting membrane. × 5600.

FIG. 11a, b.—Transverse sections of nerve fibres from the distal branches of sciatic nerve, showing Wallerian degeneration. The specimen was obtained twenty days after tumour inoculation. Note that both the axon and myelin sheath are disintegrating. Figure 11b is an advanced stage of this process. × 1400.

1

2

3

4

5

Kashef and Gupta.

6

7

Kashef and Gupta.

8

9

10

11a

11b

In the final stage, the Schwann cell cytoplasm in the affected segments contained fragmented myelin debris. Most macrophages seen contained degraded myelin and lipid droplets (Fig. 10). In macrophages the myelin debris was always surrounded by a distinct limiting membrane, whereas this was not the case in Schwann cells (Fig. 7, 10).

In this study, there was no evidence of Wallerian degeneration occurring in the nerve in the region of contact with the tumour. Nerves removed from the foot pad in the late stages of tumour growth showed mostly normal fibres. Evidence of Wallerian degeneration was occasionally found in some of the terminal branches of the involved sciatic nerve (Fig. 11a, b).

The fibroblasts alongside the reactive Schwann cells became increasingly hypertrophied with advancing demyelination and the collagen content appeared to be increased. Mast cells were frequently seen during intermediate and final stages of demyelination. The capillaries showed that the lumen was patent at the site of maximum contact with the tumour and again no tumour cells could be identified within them.

### DISCUSSION

The presence of segmental demyelination as a morphological entity was first described by Gombault (1881) in lead neuropathy in guinea pigs. Since then this type of demyelination has been observed in a variety of neurological ailments. The observations made in this study also point to segmental demyelination as a distinct factor in peripheral neuropathy in experimentally induced tumours.

In a study of diphtheritic neuropathy in guinea pigs Webster *et al.* (1961) found no evidence of myelin breakdown in peripheral nerves until after the onset of paralysis. On the other hand, Weller (1965), studying the effect of diphtheritic neuropathy in chickens, demonstrated that myelin breakdown did occur before the onset of frank paralysis. Although the rate of growth and local spread of a malignant tumour cannot be absolutely controlled, this study demonstrates that impairment of function in peripheral nerves was by segmental demyelination. In an experimental system like ours, it is not always possible to pinpoint the earliest onset of myelin breakdown. But the presence of myelin fragmentation in rats which developed slight limps suggests that the sequence of early changes are quite similar to neuropathies studied under more controlled conditions (Cavanagh and Jacobs, 1964; Weller, 1965).

Segmental demyelination was first observed on the seventh day, when the tumour was only 0·8 cm. in maximum diameter and the skin over it was absolutely lax. Dissection of the right hind limb did not show any evidence of what could be considered as a pressure on the nerve. Furthermore, there was no apparent sign of vascular insufficiency to the nerve at this region. Consequently the view that segmental demyelination was initiated either by pressure alone or in association with vascular insufficiency seems unlikely. However, as time advanced and paralysis became more pronounced, the size of the tumour also increased. Hence in the very late stages of tumour growth, it cannot be ruled out that the pressure exerted by the tumour might enhance the process of demyelination.

In this study there was no evidence of Wallerian degeneration occurring in the region of contact with the tumour. Again, nerves from the foot pad in the late stages of the tumour growth showed mostly normal fibres and very occasional evidence of Wallerian degeneration but no segmental demyelination. If pressure alone was the only cause of palsy, then one would have noted more evidence of Wallerian degeneration in the distal part of the nerve.

The only evidence of remyelination in this study was seen in the second week after tumour inoculation. The tumour being a rapidly growing one possibly interferes with normal functioning of the Schwann cells and stops the process of remyelination in the late stage of paralysis.

The mechanisms by which the tumour cells bring about myelin breakdown is not immediately apparent. The theoretical possibility of the tumour producing a chemical, initiating demyelination, though highly improbable, is indeed difficult to dispel. On the other hand, a justifiable point could be raised that this demyelination is an expression of immune response to the tumour. In order to explore these two possibilities, sciatic nerves from the contralateral sides as well as other peripheral nerves from these animals were examined. There was no evidence of any demyelination in these nerves. Moreover, in the affected nerves no lymphocytes, transformed cells or plasma cells could be seen. Therefore, it can be stated that neither of these two possibilities played a significant role in the initiation of segmental demyelination in this study.

It is tempting to postulate that the tumour cells are acting as biological competitors to Schwann cells for survival and growth, since a malignant cell is metabolically far more active than any normal cell. The Schwann cells in these circumstances may lose the power to support the myelin sheath and the breakdown process begins. Once the process of demyelination is initiated it continues, because the tumour keeps growing and the number of malignant cells in the endoneurial space keeps increasing, and the vicious cycle continues. Of course we can only suggest the plausibility of this hypothesis rather than demonstrate it at the present stage of our observations.

Recently, carcinomatous neuropathy in man has become a subject of intensive investigation. Croft *et al.* (1965) have observed patchy loss of myelinated fibres in peripheral nerves in some cases of human carcinoma of the lung. On the basis of this observation an immune theory has been alluded to. However, in our experimental system the effect was entirely local and the primary disturbance was segmental demyelination. Since there was no evidence of any abnormality in other parts of the peripheral nervous system it is appropriate to assume that in the present study the immune mechanism was unimportant.

The clinical application of this study is apparent in so far as these experiments demonstrate that peripheral nerve paralysis due to malignant tumours is initially the result of segmental demyelination. Mechanical pressure of the growing tumours and local ischaemia do not appear to be major factors. It is most probable that the presence of malignant cells in the endoneurial space initiates a chain reaction in the Schwann cells which ultimately leads to functional imparment of the involved nerve.

### SUMMARY

The patholgical changes in the sciatic nerves of rats in the presence of Walker 256 carcinoma have been investigated. A light and electron microscopic study of the affected sciatic nerves showed that the primary disturbance was segmental demyelination. Furthermore, Wallerian degeneration was infrequent in the nerves distal to the tumour, suggesting that pressure alone is not a major factor in perpheral nerve palsy. There was no evidence of any change in the peripheral nerves in other parts of the body. This implies that an immune mechanism again is unimportant. From this study it can be concluded that peripheral nerve paralysis in the presence of malignant tumours in rats is a local phenomenon and the primary abnormality is segmental demyelination.

We are grateful to Professor Roger Warwick for his guidance and encouragement during this investigation. We also wish to thank Dr. P. L. Williams, Reader in Experimental Neurology at Guy's Hospital, and Dr. J. B. Cavanagh, Director of the M.R.C. Research Group in Applied Neurobiology, Institute of Neurology, Queen Square, for their advice and criticism. We are indebted to the National Spastics Society for the use of the electron microscope.

### REFERENCES

CAVANAGH, J. B. AND JACOBS, JEAN M.—(1964) *Br. J. exp. Path.*, **45**, 309.
CROFT, P. B., HENSON, R. A., URICH, H. AND WILKINSON, P. C.—(1965) *Brain*, **88**, 501.
GOMBAULT, A.—(1881) *Archs Neurol., Paris*, **1**, 177.
MERCER, E. H. AND EASTY, G. C.—(1961) *Cancer Res.*, **21**, 52.
PALADE, G. E.—(1952) *J. exp. Med.*, **95**, 285.
WEBSTER, H. DE F., SPIRO, D., WAKSMAN, B. AND ADAMS, R. D.—(1961) *J. Neuropath. exp. Neurol.*, **20**, 5.
WELLER, R. O.—(1965) *J. Path. Bact.*, **89**, 591.